# The Roles of Glutamine in the Intestine and Its Implication in Intestinal Diseases

**DOI:** 10.3390/ijms18051051

**Published:** 2017-05-12

**Authors:** Min-Hyun Kim, Hyeyoung Kim

**Affiliations:** 1 Food Science and Human Nutrition Department, Center for Nutritional Sciences, College of Agricultural and Life Sciences, University of Florida, Gainesville, FL 32611, USA; mhkim27@ufl.edu; 2 Department of Food and Nutrition, Brain Korea 21 PLUS Project, College of Human Ecology, Yonsei University, Seoul 03722, Korea

**Keywords:** glutamine, intestinal function, inflammatory bowel disease, short bowel syndrome, nutritional therapy

## Abstract

Glutamine, the most abundant free amino acid in the human body, is a major substrate utilized by intestinal cells. The roles of glutamine in intestinal physiology and management of multiple intestinal diseases have been reported. In gut physiology, glutamine promotes enterocyte proliferation, regulates tight junction proteins, suppresses pro-inflammatory signaling pathways, and protects cells against apoptosis and cellular stresses during normal and pathologic conditions. As glutamine stores are depleted during severe metabolic stress including trauma, sepsis, and inflammatory bowel diseases, glutamine supplementation has been examined in patients to improve their clinical outcomes. In this review, we discuss the physiological roles of glutamine for intestinal health and its underlying mechanisms. In addition, we discuss the current evidence for the efficacy of glutamine supplementation in intestinal diseases.

## 1. Introduction

Glutamine is the most abundant amino acid in human blood, skeletal muscle, and the free amino acid pool [1]. It plays physiologically important roles in various metabolic processes: as an intermediary in energy metabolism, and as a substrate for the synthesis of peptides and non-peptides such as nucleotide bases, glutathione, and neurotransmitters [2,3,4]. Additionally, glutamine contributes to the detoxification of ammonia and systemic acid-base balance [5]. The involvement of glutamine metabolism in immune systems [1,6,7] and in cancer cells [8,9,10] has been documented in the past two decades. Moreover, glutamine metabolism has direct relevance to clinical medicine. This was initially highlighted when glutamine, classically a non-essential amino acid, was considered to be conditionally essential during certain catabolic states, such as trauma or sepsis [11]. The theory of “conditionally essential” glutamine during illness was based on observations that intestinal, renal, and immune cells utilize large amounts of glutamine, exceeding the endogenous glutamine production [12,13], and that plasma and muscle glutamine levels are markedly reduced in these conditions [14].

Among the various tissues using glutamine at high rates, the intestine utilizes about 30% of total glutamine [15], indicating that it is a key nutrient for the intestine. Studies in healthy adults have demonstrated that three quarters of enterally provided glutamine is absorbed into the splanchnic tissues, and most of the absorbed glutamine is metabolized within the intestine [16,17]. One-fourth of the plasma glutamine is taken up by the small intestine when it passes the organ [18]. It has been reported that the intestine competes with other tissues for glutamine from the body amino acid pool and dietary sources [19]. Glutamine metabolism in the intestine has been intensively studied. Its functions include maintaining nucleotide metabolism and intestinal barrier function, modulation of inflammation, and regulating stress responses and apoptosis [20,21,22]. Concomitantly, the efficacy of glutamine supplementation has been tested in humans and animal models with intestinal diseases [23,24,25]. This review aims to discuss the role of glutamine in the intestine, and to summarize current evidence for the clinical efficacy of glutamine supplementation in intestinal diseases, especially inflammatory bowel diseases (IBDs).

## 2. Roles of Glutamine in the Intestine

### 2.1. Tissue Integrity

The mammalian intestinal lumen is lined with a single layer of epithelial cells [26]. As these cells are renewed every four to five days, a continuously high level of cell proliferation is required to maintain homeostasis [27]. In general, cell proliferation is regulated by a number of signaling pathways and hormones such as growth factors. When proliferation is activated by these signals, crypt-residing intestinal stem cells differentiate into specialized epithelial cell types, including enterocytes, goblet cells, paneth cells, and enterocytes, which enables the maintenance of normal intestinal tissue integrity [28].

**Figure 1:**
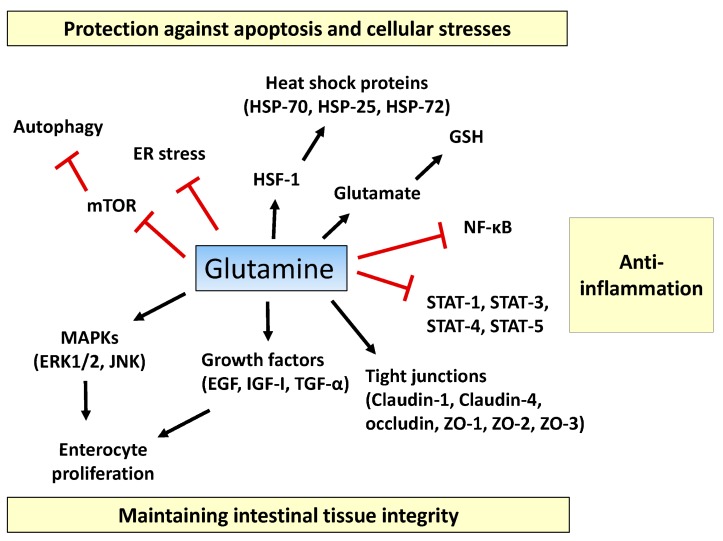
Proposed action mechanisms of glutamine in intestinal cells. Glutamine maintains intestinal tissue integrity via promoting enterocyte proliferation, activation of mitogen-activated protein kinases (MAPKs) (ERK1/2, JNK1/2), optimizing the actions of growth factors (epidermal growth factor (EGF), insulin-like growth factor (IGF)-I, transforming growth factor (TGF)-α), and inducing expression of tight-junction proteins (claudin-1, claudin-4, occludin, zonula occludens (ZO)-1, ZO-2, and ZO-3). Pro-inflammatory signaling pathways such as the nuclear factor-κB (NF-κB) and signal transducers and activators of transcription (STAT) pathways are inhibited by glutamine. Glutamine suppresses extensive apoptosis by participating in the synthesis of glutathione (GSH) and by regulating heat shock factor (HSF)-1-mediated expression of heat shock proteins (HSPs). Glutamine ameliorates endoplasmic reticulum (ER) stress and promotes autophagy by inhibiting the mechanistic target of rapamycin (mTOR) pathway, thus protecting intestinal cells from stressful conditions. T bars mean inhibition while arrows represent stimulation.

Glutamine influences a number of signaling pathways that regulate cell cycle regulation and proliferation. Mitogen-activated protein kinases (MAPKs) are protein kinases that orchestrate a number of cell functions, including cell proliferation and differentiation [29]. Rhoads et al. demonstrated that glutamine is required for intestinal cell proliferation by activating multiple MAPKs, including extracellular signal-regulated kinases (ERK1/2) and c-Jun N-terminal kinases (JNK1/2), in the rat intestinal mucosal cell line, IEC-6 [30] (Figure 1). Additionally, glutamine contributes to intestinal cell proliferation by augmenting the effects of growth factors such as epidermal growth factor (EGF), insulin-like growth factor-I (IGF-I), and transforming growth factor-α (TGF-α). Restriction of glutamine in cell culture media resulted in impaired EGF-stimulation of DNA, RNA, and protein synthesis and cell replication in IEC-6 cells [31]. Consumption of glutamine-enriched diets significantly enhanced IGF-I-mediated DNA and protein synthesis in a rat model of short bowel syndrome [32]. In a porcine model of ischemia, glutamine administration enhanced the action of TGF-α on mucosal cell proliferation [33].

Tight junctions, consisting of various proteins, seal adjacent epithelial cells to produce a physical barrier between epithelial and endothelial cells [34]. Tight junctions are dynamic rather than static structures. Indeed, tight junctions constantly remodel their structures with a relatively high rate of turnover to interact with external stimuli by which they control the entry of ions, nutrients, and water [35]. In addition, tight junctions maintain intestinal integrity, which prevents pathogens and toxins from entering the intestinal lumen [36]. There are four types of transmembrane components of tight junctions, including claudins, occludin, tricellulin, and junctional adhesion molecules [37]. In response to various physiological stimuli and signal pathways, tight junctions modulate the transport of luminal molecules into mucosal cells by adjusting their tightness [38]. These signaling molecules are protein kinase C, MAPKs, and myosin light chain kinases (MLCK). Activation of protein kinase C resulted in the upregulation of occludin, zonula occudens (ZO)-1, ZO-2, and claudin 1 in primary human epithelial cells, leading to enhancing transepithelial electrical resistance [39]. MAPKs could directly interact with the C-terminal tail of occludin, which mediates the prevention of hydrogen peroxide-induced disruption of tight junctions [40]. Furthermore, MLCK-induced phosphorylation of myosin light chain regulated tight junction permeability in Caco-2 cells [41,42].

Multiple lines of evidence indicate that glutamine modulates the expression of tight junction proteins. In the human colon carcinoma cell line Caco-2, glutamine deprivation markedly reduced the expression of multiple tight junction proteins, including claudin-1, occludin, and ZO-1 [43]. Restriction of glutamine in cell culture media significantly increased epithelial cell permeability in Caco-2 cells, as determined by multiple methods including transepithelial electrical resistance (TER), tracer models using ^14^C-mannitol, and fluorescein isothiocyanate-dextran [44]. The addition of glutamine in glutamine-deprived cells rescued the impaired barrier functions. In methotrexate-treated Caco-2 cells, the addition of glutamine improved permeability along with increased ZO-1 and occludin expressions [45]. By measuring TER and inulin flux, Seth et al. also reported that the addition of glutamine prevented acetaldehyde-induced disruption of tight junctions and impaired paracellular permeability [46]. Maintaining intestinal permeability by tight junction proteins has shown to be beneficial for treatment of multiple intestinal pathologic conditions such as inflammatory bowel disease and celiac disease [47]. These studies suggest that glutamine supplementation may be beneficial for individuals with an impaired gut permeability by enhancing the expression of tight junction proteins.

Mechanistically, glutamine has been shown to influence a number of signaling pathways that regulate the expression of tight junction components [48], although much of this process still remains unclear. In the Caco-2 cells, deprivation of glutamine activated the phosphatidylinositol 3-kinase (PI3K)/Akt pathway, which led to a reduction in claudin-1 expression and TER [49]. On the contrary, glutamine supplementation reduced the activation of the PI3K/Akt pathway, which reversed claudin-1 expression in the cells, suggesting that glutamine supplementation regulates phosphorylation states of tight junction proteins. Interestingly, normal formation of tight junction is controlled by phosphorylation of occludin and ZO-1 [50,51,52]. Therefore, glutamine-mediated tight junction maintenance is in part mediated by phosphorylation of tight junction proteins. Wang et al. showed that glutamine activated calcium/calmodulin-dependent kinase 2-AMP-activated protein kinase signaling in porcine jejunal enterocytes [53]. They also reported that glutamine induced the expression of ZO-1, ZO-2, and ZO-3 and caused greater distribution of claudin-1, claudin-4, and ZO-1 at the plasma membranes [53]. Dokladny et al. reported that heat shock-induced activation of heat shock factor-1 (HSF-1) induced nuclear translocation of HSF-1 and expression of occluding in Caco-2 cells [54].

### 2.2. Inflammatory Pathway

Inflammation has been shown to be a cause of intestinal diseases, such as ulcerative colitis, Crohn’s disease, and colorectal cancer [55]. Therefore, treatment of intestinal inflammation is important to target these diseases. Several lines of evidence indicate that glutamine has an anti-inflammatory property by influencing a number of inflammatory signaling pathways, including the nuclear factor κB (NF-κB) and signal transducer and activator of transcription (STAT) pathways [56].

The transcription factor NF-κB regulates a number of immune responses [57]. During the early phase of infection, it activates various genes to boost the inflammatory reaction. NF-κB is a multiprotein complex composed of five members of the Rel family: p50, p52, p65, RelB, and c-Rel. Under steady-state conditions, NF-κB resides in the cytoplasm and is maintained inactive by a family of inhibitors, designated inhibitor of κB (IκB). In response to various extracellular stimuli, IκB kinase phosphorylates IκB proteins, triggering their degradation and release from NF-κB, which thus becomes active. Active NF-κB complex is translocated into the nucleus where it induces the expression of genes harboring NF-κB-binding elements, such as *interleukin-6* (*Il-6*) and *tumor necrosis factor*-*α* (*Tnf-α*). During inflammatory status, productions of inflammatory cytokines IL-6 and TNF-α are highly elevated, which stimulates immune response by activating multiple target cells such as antigen-presenting cells and T cells and by inducing acute-phase proteins [58]. Glutamine has been shown to suppress NF-κB pathway activation. Administration of glutamine via intraperitoneal injection or oral gavage suppressed NF-κB activation in rodent models of colitis [59,60] and in lipopolysaccharide (LPS)-treated piglet enterocytes [61]. Mechanistically, NF-κB suppression by glutamine was associated with increased expression of cellular heat shock proteins (HSPs) such as HSP25 and HSP70, which are induced by HSF-1 [60]. The HSF-1-mediated heat shock response has been shown to inhibit NF-κB activation and NF-κB-dependent gene expression. This hypothesis was supported by a marked increase in NF-κB activation in embryonic fibroblast from HSF-1-null mutant mice due to lack of HSPs expression [62]. These studies suggest that the anti-inflammatory effect of glutamine, in part, may be related to HSF-1 activation and suppression of NF-κB-mediated inflammatory cytokine expression. Additionally, glutamine influences IκB stability. In LPS-treated Caco-2 cells, deprivation of glutamine reduced the expression of IκBα, triggering elevated NF-κB binding to DNA as well as increased expression of inflammatory cytokine interleukin-8 (IL-8) [63]. In human ileocecal adenocarcinoma HCT-8 cells, pre-treatment with glutamine reduced the level of IκBα degradation and production of IL-8 during TNF-α-induced inflammation [64]. In support of these findings in vitro, Kretzmann et al. showed that glutamine supplementation for seven days significantly reduced IκBα degradation, leading to suppression of NF-κB activation in a rat colitis model [65]. Since production of IL-8, a cytokine that stimulates migration of neutrophils to inflammatory sites, was influenced by glutamine status [63,64], glutamine-mediated IL-8 regulation could be an important event for targeting intestinal inflammation.

STAT proteins are transcription factors that modulate the immune system, cellular proliferation, and development [66]. They have been extensively studied for their roles in regulating inflammation by inducing the expression of cytokines including IL-6 [67]. In rat colitis models, glutamine administration via the rectal route reduced the phosphorylation of STAT1 and STAT5, indicating that glutamine influences STAT signaling activation [65]. In LPS-treated Caco-2 cells, glutamine depletion upregulated STAT4, whereas glutamine supplementation downregulated STAT4 expression and IL-8 production [68]. Therefore, the anti-inflammatory effect of glutamine may be contributed to by inhibiting STAT activation and by inhibiting expression of inflammatory cytokines such as IL-6 and IL-8 in intestinal tissues.

Nitric oxide (NO) is synthesized by multiple cells and modulates a variety of cellular signaling pathways, including inflammatory responses [69]. During intestinal inflammation, NO may play a dichotomous role, as both beneficial and harmful effects of NO have been observed [70]. Glutamine is an important regulator of NO synthesis [71,72]. Houdijk et al. reported that the level of whole body plasma nitrate, the stable end-product of NO production, was reduced in rats fed a glutamine-enriched diet [73]. Similarly, in rats with intestinal ischemia-reperfusion injury, glutamine-enriched diet reduced mucosal expression of inducible NO synthase, an inflammatory enzyme, and decreased the plasma NO concentration [74].

Since sustained activation of inflammatory signaling pathways and prolonged production of pro-inflammatory cytokines are critical in the development and progress of intestinal inflammatory diseases, there has been efforts to suppress the production of inflammatory mediators to treat patients with intestinal inflammatory diseases including IBD [75]. Therefore, based on the in vitro and in vivo studies mentioned above, glutamine supplementation could be one promising candidate for treating intestinal inflammatory disorders by inhibiting activation of NF-κB and STAT, and suppressing expression of inflammatory cytokines such as IL-6, TNF-α, and IL-8, and inflammatory enzyme inducible NO synthase.

### 2.3. Apoptosis and Cellular Stresses

As intestinal epithelial cells have a turnover rate of four to five days, it is critical for these cells to maintain a fine balance between proliferation and apoptosis for normal function [76]. Spontaneous apoptosis in intestinal epithelia is essential for maintaining its normal architecture [77]. However, a number of cellular stresses induced by exogenous agents or by intracellular stimuli including endotoxemia, nutrient deprivation, and lack of growth factor can disturb the balance between proliferation and apoptosis. This imbalance between proliferation and apoptosis triggers intestinal pathologic conditions due to sustained apoptotic cell death [78,79,80,81]. Indeed, loss of epithelial cells in ulcerative colitis and bacterial infection mainly occurs by increased apoptosis in crypts [82,83]. Intestinal inflammatory disorders such as celiac disease and nematode infections are highly associated with increased apoptosis of intestinal epithelial cells [84,85]. Even though the differentiated enterocytes undergo apoptosis which maintains normal gut epithelial function, dysregulated apoptosis is seen in a number of pathological conditions in the gastrointestinal tract. Therefore, it is critical to inhibit apoptosis of intestinal epithelial cells to prevent the intestinal pathologic conditions [86]. Glutamine has been shown to display anti-apoptotic properties in intestine. In rat intestinal epithelial (RIE-1) cells, glutamine deprivation resulted in apoptosis [87]. Similarly, glutamine supplementation effectively reduced toxin-induced apoptosis in human intestinal epithelial T84 cells [88], and sodium laurate-induced apoptosis in RIE-1 cells [89], collectively suggesting that glutamine is critical to suppressing apoptosis.

Further studies have demonstrated the mechanisms underlying the anti-apoptotic capacity of glutamine. First, as a precursor for glutathione (GSH), glutamine prevents apoptosis by maintaining normal cellular redox status. Along with cysteine and glycine, glutamate converted from glutamine produces GSH, an important cellular antioxidant [90]. GSH is present in the cell in both reduced and oxidized forms (GSSG), and the ratio of GSH to GSSG determines the cellular redox potential. Because depletion of GSH induces apoptosis due to excessive oxidative stress [91], normal glutamine metabolism plays a critical role in preventing apoptosis by providing glutamate required for maintaining normal GSH/GSSG ratio.

Secondly, glutamine regulates caspase activation. Caspases are a family of protease enzymes that play important roles in inducing apoptosis [92]. Basically, they are present as a proenzyme, but various stimuli can activate the enzymes through cleavage. In RIE-1 cells, glutamine-deprived cells showed significantly higher caspase-3 activity along with a higher level of apoptosis [93]. Administration of glutamine reduced caspase-3 activity in neonatal piglet enterocytes [61] as well as caspase-8 activity in T84 cells [88].

In addition, glutamine enhances the expression of heat shock proteins (HSPs) [94]. HSPs have been reported to modulate apoptotic cell death by acting as a molecular chaperone, allowing the cells to adapt to stressful conditions [95]. In rats with sepsis, glutamine administration significantly increased the expression of HSP-70 and HSP-25, possibly via increased phosphorylation of heat shock factor (HSF)-1 [96]. The increased expression of HSPs via glutamine supplementation markedly improved the survival rate of rats with sepsis. Ropeleski et al. reported that glutamine enhanced HSF-1-mediated gene expression of *Hsp-72* during heat shock in IEC-18 and H4 intestinal cells [97]. On the contrary, glutamine-deficient conditions reduced gene expression of *Hsp-72*, which resulted in higher caspase-3 activity and apoptotic cell death.

The protective effect of glutamine is also attributed to its role in modulating cellular stress responses such as endoplasmic reticulum (ER) stress and autophagy. The ER is an organelle responsible for protein synthesis, folding, and modification. A number of pathologic conditions, including IBD, disrupt ER function, resulting in ER stress [98]. As extensive ER stress triggers sustained apoptosis and further insults, attenuating ER stress is critical for cell protection and survival. In rats with colitis, administration of glutamine markedly reduced the activation of ER stress markers, such as glucose responsive protein 78, CCAAT/enhancer binding protein (C/EBP) homologous protein, and caspase-12 [99]. These results show that glutamine supplementation reduces ER stress and apoptosis. Supporting these observations, glutamine treatment reduced the activation of ER stress in Caco-2 cells treated with pharmacological ER stress inducers.

Autophagy is a catabolic process activated during a number of metabolic stress conditions, such as nutrient deprivation [100]. Upon activation, autophagy breaks down cellular organelles and proteins to supply them as an energy source. It has been shown that autophagy provides a protective effect against intestinal pathologic conditions. *Autophagy-related 16-like 1* (*Atg16L1*), a gene essential for functional autophagosome, has been implicated in Crohn’s disease [101,102]. Saitoh et al. showed that mice lacking *Atg16L1* were more susceptible to induced acute colitis, supporting the importance of autophagy to inhibit intestinal inflammation [103]. Furthermore, mutation of *Atg5* and *Atg7*, autophagy-related genes, in mouse intestinal epithelium resulted in increased production of TNF-α and IL-1β following LPS administration [104]. Additionally, Paneth cells lacking *Atg16L1*, *Atg5*, or *Atg7* showed impaired secretion of antimicrobial proteins, which confer intestinal protection against pathogens [105]. Since IBD pathogenesis is associated with dysfunction of Paneth cells [106], functional autophagy is essential for intestinal homeostasis and preventing intestinal inflammation. Glutamine has been reported to increase autophagy in intestinal epithelial cells. In Caco-2 and IEC-18 cells, Sakiyama et al. reported that glutamine treatment augmented the number of autophagosomes as well as the level of microtubule-associated protein light chain 3-phospholipid conjugates, which are markers for autophagy activation [107]. As a result, the enhanced autophagy via glutamine treatment suppressed intestinal apoptosis under stress conditions. The authors demonstrated that glutamine regulates autophagy by influencing mechanistic target of rapamycin (mTOR) signaling. The mTOR pathway integrates signals from nutrients status and growth factors to modulate multiple cellular processes, including autophagy [108]. In agreement with this finding, Van Der Vos et al. demonstrated that glutamine synthetase is a target of forkhead box O3 (FOXO3), which is a transcription factor activated during autophagy [109]. FOXO3-mediated glutamine synthesis resulted in mTOR inhibition, which promoted autophagy activation and cellular survival.

## 3. Clinical Implications for Intestinal Diseases

Given the importance of glutamine in maintaining normal cellular functions, as discussed above, it is not surprising that glutamine supplementation has been considered and examined in the clinical setting, particularly in diseases implying impaired glutamine metabolism. A conditional glutamine deficient status has been postulated in patients with acute critical illness. In these patients, marked reduction in plasma glutamine concentration is possibly due to the consequence of muscle wasting [14]. Regarding intestinal diseases, patients with Crohn’s disease display low plasma and cellular glutamine concentrations, and reduced mucosal glutaminase activity [110]. These observations led to the hypothesis that glutamine supplementation would improve clinical outcomes.

A number of experiments in animals with IBD demonstrated that glutamine supplementation is able to protect the intestinal mucosa, raising the possibility of use of glutamine support in human patients. In mice with dextran sulfate sodium-induced colitis, oral glutamine supplementation (41.7 g/kg diet; 10 days) resulted in mitigated colonic inflammatory reactions [111] as well as increased expression of small-intestinal intraepithelial γδ-T cells [112]. In dextran sulfate sodium-induced rats, administration of glutamine (0.75 g/kg body weight (BW)/d; 7 days) by oral gavage increased HSP25 and HSP70, and reduced bleeding and diarrhea [60]. Rats with trinitrobenzene sulfonic acid-induced colitis that received dietary glutamine supplementation (20 g/kg or 40 g/kg; 2 weeks) showed reduced production of pro-inflammatory cytokines, including TNF-α and IL-8, bacterial translocation, and inflamed lesions [113]. Oral glutamine supplementation (3% in drinking water) ameliorated abdominal radiation-induced mucosal injury and reduced bacterial translocation in gut mucosa of rats [114]. Injection of glutamine (0.75 g/kg BW) in mice with sepsis model ameliorated sepsis-induced inflammatory reactions by modulating intestinal intraepithelial lymphocytes [115,116].

Based on these positive results in animal models, human studies have been conducted in an attempt to support the efficacy of glutamine supplementation in improving disease status. However, only a limited number of studies concluded that glutamine supplementation has a beneficial effect in intestinal diseases. In a systematic review by García-de-Lorenzo et al., glutamine-enriched diets were shown to improve immunologic aspects in trauma patients and to ameliorate mucositis in post-chemotherapy patients [117]. The authors determined how much glutamine is required to observe improved clinical outcomes: 21 g glutamine/day for 28 days for Crohn’s disease, and 42 g/day for 21 days for short bowel syndrome. In a randomized controlled trial, Benjamin et al. reported that glutamine supplementation (0.5 g/kg BW; 2 months) in patients with Crohn’s disease in remission phase reduced the intestinal permeability and morphology [118].

However, a number of studies did not observe any improved outcomes from glutamine supplementation. In recent randomized and controlled trails, termed Scottish Intensive Care Glutamine or Selenium Evaluative Trial (SIGNET), effects of a parental administration of glutamine (0.1 to 0.2 g/kg BW/day) were evaluated in 500 patients with critical illness [119]. However, the SIGNET study did not show any beneficial outcome of glutamine supplementation. More recently, Reducing Death Due to Oxidative Stress (REDOXS), a randomized and controlled trial, examined glutamine supplementation (0.6 to 0.8 g/kg BW/day) in 1223 critically ill patients [120]. Not only was there no effect of glutamine supplementation on rates of organ failure or infectious complication, but also patients who received enteral glutamine treatment showed a trend toward elevated rate of death at 6 months. Similarly, glutamine supplementation on intestinal diseases do not clearly support efficacy of glutamine supplementation. Akobeng et al. examined the effect of glutamine-enriched polymeric diet (8 g/day for 4 weeks) in 18 children with active Crohn’s disease, and found no changes in parameters tested, including intestinal permeability [121]. Similarly, no significant effect of oral glutamine supplements (21 g/day for 4 weeks) was observed in 14 Crohn’s disease patients [122]. In short bowel syndrome patients, six studies have examined the effect of glutamine support, but no improvement in the surrogate parameters was found [123,124,125,126,127,128]. Although some studies showed favorable effects, the clinical efficacy of glutamine supplementation in intestinal diseases remains a controversial issue.

Alpers et al. pointed out limitations of current clinical studies on glutamine supplementation [129], providing some clues for these inconsistent results. The first thing to consider is whether it is true that humans are really glutamine deficient during the diseases. The “conditionally essential glutamine” theory is still a prediction and is uncertain. Because the levels of amino acids including glutamine dynamically and constantly are changed in tissues and plasma, and plasma levels do not always reflect tissue levels, it is challenging to determine whether glutamine is conditionally essential. In addition, the reduced plasma concentration during critical illness is not specific for glutamine, but it occurs for most amino acids [129]. Parenteral glutamine supplementation in critically ill patients did not restore muscle glutamine depletion of the patients, which raises a question for glutamine deficiency in the patients [130]. Furthermore, plasma glutamine level in seriously ill patients did not predict mortality of the patients [131], suggesting that plasma glutamine status may not be correlated with severity of disease. Therefore, an effort to validate a glutamine deficiency state in the pathologic conditions should be preceded before predicting clinical efficacy of glutamine supplementation.

Various experimental designs could have influenced the indefinite results of clinical studies. First, glutamine was administered in two different ways: total parental nutrition and enteral nutrition. Generally, it is stated that enteral nutrition is safer for prolonged period than parental nutrition, whereas parental nutrition is often recognized as being better for achieving targeted calorie requirement, especially in critically ill patients [132]. Route of administration influences the contribution of glutamine [133]. In patients with acute ulcerative colitis, total enteral nutrition was shown to be nutritionally effective as well as produce fewer complications compared to enteral nutrition [134]. Given total parenteral nutrition produces changes in intestinal morphology and function [135], glutamine supplementation via parenteral nutrition might cause complications in intestine. Second, a wide variety of dose, time, and mode of supplementation was used. Dose of glutamine used in the studies varied up to 5-fold [121,122,136], and treatment period varied from 2 days [124] to 8 weeks [128]. As glutamine couples with alanine and glycine, glutamine complex with alanine and glycine is less susceptible to degradation than free glutamine. Many studies used glutamine-containing dipeptides, which might affect the discrepancy of outcomes. Third, a wide range of clinical courses of patients was used in the studies. Short-term glutamine administration during a flare-up phase could give a greater impact on outcomes than other phases. Additionally, a relatively small sample size showed greater efficacy of glutamine supplementation in critically ill patients [137,138,139] and in IBD patients [117,118]. Therefore, a well-controlled clinical trial with a sufficiently sized population would be required to determine the efficacy of glutamine supplementation in intestinal diseases.

## 4. Conclusions

In this review, we covered the roles of glutamine in the intestine, including the regulation of enterocyte proliferation, maintenance of tight-junction proteins, modulation of inflammatory pathways, such as NF-κB and STAT signaling, and protection against apoptosis and cellular stresses, which are summarized in Figure 1. Although significant progress has been made in uncovering the functions of glutamine, most of these are based on observational studies. Therefore, future research should focus on the mechanisms underlying glutamine actions. Additionally, current data from clinical trials do not support the use of glutamine supplementation in patients with intestinal diseases, despite in vitro and animal model studies having shown significant beneficial effects. Thus, future human studies should be more standardized to increase their power.
